# Prediabetes and major adverse cardiac events after acute coronary syndrome: An overestimated concept

**DOI:** 10.1002/clc.24262

**Published:** 2024-04-01

**Authors:** Amir Hossein Behnoush, Saba Maleki, Alireza Arzhangzadeh, Amirmohammad Khalaji, Parmida Sadat Pezeshki, Zahra Vaziri, Zahra Esmaeili, Pouya Ebrahimi, Haleh Ashraf, Farzad Masoudkabir, Ali Vasheghani‐Farahani, Kaveh Hosseini, Mehdi Mehrani, Adrian V. Hernandez

**Affiliations:** ^1^ Cardiac Primary Prevention Research Center, Cardiovascular Diseases Research Institute Tehran University of Medical Sciences Tehran Iran; ^2^ Tehran Heart Center, Cardiovascular Diseases Research Institute Tehran University of Medical Sciences Tehran Iran; ^3^ School of Medicine Tehran University of Medical Sciences Tehran Iran; ^4^ Non‐Communicable Diseases Research Center, Endocrinology and Metabolism Population Sciences Institute Tehran University of Medical Sciences Tehran Iran; ^5^ School of Medicine Guilan University of Medical Sciences (GUMS) Rasht Guilan Province Iran; ^6^ Department of Cardiology, School of Medicine Shiraz University of Medical Sciences Shiraz Iran; ^7^ Student Research Committee Babol University of Medical Sciences Babol Iran; ^8^ Jundishapur University of Medical Sciences Ahvaz Iran; ^9^ Health Outcomes, Policy, and Evidence Synthesis (HOPES) Group University of Connecticut School of Pharmacy Storrs Connecticut USA; ^10^ Unidad de Revisiones Sistemáticas y Meta‐análisis (URSIGET), Vicerrectorado de Investigación Universidad San Ignacio de Loyola Lima Peru

**Keywords:** acute coronary syndrome, diabetes, percutaneous coronary intervention, prediabetes

## Abstract

**Background:**

Unlike diabetes, the effect of prediabetes on outcomes in patients with acute coronary syndrome (ACS) who underwent percutaneous coronary intervention (PCI) is not much investigated. We investigated the association between fasting glycemic status and major adverse cardiovascular and cerebrovascular events (MACCE) in patients with ACS undergoing PCI and had mid to long‐term follow‐up after coronary stenting.

**Methods:**

Registry‐based retrospective cohort study included ACS patients who underwent PCI at the Tehran Heart Center from 2015 to 2021 with a median follow‐up of 378 days. Patients were allocated into normoglycemic, prediabetic, and diabetic groups. The primary and secondary outcomes were MACCE and its components, respectively. Unadjusted and adjusted Cox models were used to evaluate the association between glycemic status and outcomes.

**Results:**

Among 13 682 patients, 3151 (23%) were prediabetic, and 5834 (42.6%) were diabetic. MACCE risk was significantly higher for diabetic versus normoglycemic (adjusted hazard ratio [aHR]: 1.22, 95% confidence interval [CI]: 1.06–1.41), but nonsignificantly higher for prediabetic versus normoglycemic (aHR: 0.95, 95% CI: 0.78–1.10). All‐cause mortality risk was significantly higher in diabetic versus normoglycemic (aHR: 1.42, 95% CI: 1.08–1.86), but nonsignificantly higher for prediabetic versus normoglycemic (aHR: 1.15, 95% CI: 0.84–1.59). Among other components of MACCE, only coronary artery bypass grafting was significantly higher in diabetic patients, and not prediabetic, compared with normoglycemic.

**Conclusions:**

Prediabetic ACS patients undergoing PCI, unlike diabetics, are not at increased risk of MACCE and all‐cause mortality. While prediabetic patients could be regarded as having the same risk as nondiabetics, careful consideration to provide more intensive pre‐ and post‐PCI care in diabetic patients is mandatory.

## Graphical abstract

Based on a cohort of 13 682 patients with acute coronary syndrome (ACS) who underwent percutaneous coronary intervention (PCI), prediabetes was not associated with a higher rate of adverse events; however, diabetes significantly increased the rates of major adverse cardiac and cerebrovascular events (MACCE) and all‐cause mortality.

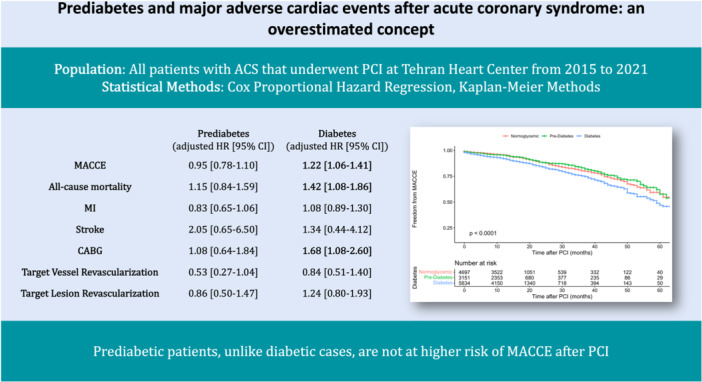

## 1 INTRODUCTION

Coronary artery disease (CAD) describes a variety of clinical presentations, from chronic coronary syndrome (CCS) to acute coronary syndrome (ACS) and seems to be a major cause of morbidity and mortality among patients suffering from diabetes.^1^, ^2^, ^3^, ^4^ The overall prevalence of diabetes has dramatically increased over the past decades worldwide, with a global population of 529 million adults worldwide, and it is estimated that the population of diabetic patients will approach 1.31 billion worldwide by 2050.^5^ Type 2 diabetes mellitus (DM) is a proven risk factor for atherosclerotic vascular disease, including CAD, peripheral arterial disease, and cerebrovascular disease.^6^ Studies have shown that about 32% of the DM population is affected by cardiovascular disease, specifically coronary events. Hence, intensive medical and invasive strategies continue to be the standard of care in DM patients.

Although for DM patients requiring revascularization, the optimal strategy remains controversial, a large number of these patients undergo percutaneous coronary intervention (PCI) in acute or chronic settings. Aside from the well‐established advantages of PCI, diabetes is considered to be an independent prognostic factor of poor outcomes in patients with CAD undergoing percutaneous revascularization.^6^, ^7^, ^8^, ^9^, ^10^ According to the European Society of Cardiology (ESC) guideline, about 25% of revascularization therapies, including PCI and coronary artery bypass graft surgery (CABG), are performed on patients with diabetes.^11^ Individuals with DM undergoing PCI are associated with worse outcomes, such as stent restenosis and major adverse cardiovascular events, compared to the normoglycemic (NG) population.^12^, ^13^, ^14^

The latest version of the American Diabetes Association (ADA) defines prediabetes as a glycosylated hemoglobin (HbA1c) level between 5.7% and 6.4% or fasting plasma glucose (FPG) level between 100 and 125 mg/dL.^15^ Prediabetic patients have also shown a positive association with CAD, increasing by two‐fold compared to the NG population,^16^, ^17^, ^18^ but the link between prediabetic CAD patients and poor outcomes remains unclear.^18^, ^19^, ^20^, ^21^, ^22^, ^23^

Previous studies have demonstrated conflicting results regarding the difference between prediabetic patients and diabetic patients and whether they are different from the normal population. While some had suggested no difference between prediabetic cases and NG groups,^24^, ^25^ some had found an increased risk in these patients.^26^, ^27^ These studies were mainly limited by small sample sizes. Moreover, they mainly did not report the data for ACS patients separately as a group with a higher risk of adverse events. Hence, herein, we explored the correlation between glycemic status, determined by FPG and mean HbA1c levels, and major adverse cardiac and cerebrovascular events (MACCE) in ACS patients undergoing PCI, categorizing baseline glycemic status as NG, prediabetic, and diabetic.

## 2 METHODS

### 2.1 Study design and population

In this registry‐based cohort study, we included patients who presented with ACS and underwent PCI at the Tehran Heart Center from 2015 to 2021. The median follow‐up time was 378 days (interquartile range: 313–589 days). ACS patients were either presented with ST‐elevation myocardial infarction (STEMI) or non‐ST elevation ACS (NSTE‐ACS). Due to the retrospective nature of our study, an “informed consent waiver” was obtained from the ethics committee, and the study proposal received approval from the ethics committee at the Tehran University of Medical Sciences (IR.TUMS.MEDICINE.REC.1402.434).

We excluded patients who had no follow‐up or no data for defining glycemic status at baseline (past medical of diabetes, FPG, or HbA1c). Moreover, diagnosis of ACS was based on the most recent guidelines and we excluded patients with CCS or nonobstructive angina.

### 2.2 Definitions and outcomes

DM was defined as FPG ≥ 126 mg/dL, HbA1c ≥ 6.5%, or a past medical history of DM. Moreover, prediabetes (pre‐DM) was defined as FPG 100–125 mg/dL, or HbA1c 5.7%–6.4%.^28^ Patients not fulfilling any of these two criteria were considered NG. Baseline characteristics recorded were demographics (age, sex, body mass index [BMI], and waist circumference [WC]), left ventricular ejection fraction (LVEF), past history of metabolic diseases (hypertension, diabetes, and dyslipidemia), cardiovascular disease history (heart failure, atrial fibrillation, valvular heart disease, peripheral vascular disease, previous PCI, previous CABG, stroke, STEMI, NSTEMI, unstable angina [UA], and stable angina [SA]), chronic lung disease history, history of cigarette smoking and opium use, family history of CAD, laboratory tests (triglycerides, total cholesterol, high‐density lipoprotein cholesterol [HDL‐C], low‐density lipoprotein cholesterol [LDL‐C], FPG, creatinine, and hemoglobin), and PCI characteristics (target lesion length, ACS type [STEMI, NSTE‐ACS], preprocedure thrombolysis in myocardial infarction flow, vessel severity [single‐vessel disease, two‐vessel disease, three‐vessel disease], ACC/AHA category of the target lesion, and PCI location).

Primary outcomes were a composite of MACCE (i.e., all‐cause mortality, myocardial infarction [MI], stroke, target vessel revascularization [TVR], target lesion revascularization [TLR], and CABG) as used in Tehran Heart Center studies.^29^ TLR was defined as repeat PCI within the index procedure stent or 5 mm edge. In our study, TVR cases were those for whom repeat PCI was performed in the target vessel but in another site than the target lesion. Secondary outcomes were MACCE components.

### 2.3 Statistical analyses

Continuous data were reported as mean ± standard deviation (SD) while categorical variables were reported as numbers (percentage). Analysis of Variance (ANOVA) and *χ* ^2^ tests were used to compare continuous and categorical variables among NG, prediabetic, and diabetic groups, respectively.

The Kaplan–Meier method was used to model freedom from events over time, and the log‐rank test was used to compare outcome incidence among NG, prediabetic, and diabetic groups. Clinical outcomes were also compared according to gender, vessel severity, and ACS presentation (STEMI or NSTE‐ACS). The association between baseline glycemic status and MACCE or its components was assessed with unadjusted and adjusted Cox proportional hazards models, and effects on outcomes were reported as hazard ratios (HRs) and their 95% confidence intervals (CIs) between diabetic or prediabetic and NG (reference group). Three Cox models were used: Model 1 had no adjustment, Model 2 was adjusted for sex and age, and Model 3 was fully adjusted for age, sex, LVEF, ACS type (STEMI, NSTEMI, and UA), vessel severity, TG, HDL‐C, LDL‐C, hypertension, WC, BMI, creatinine, hemoglobin, family history of CAD, smoking, opium use, past medical histories of STEMI, NSTE‐ACS (NSTEMI and UA), SA, heart failure, valvular heart disease, CVA, peripheral vascular disease, chronic lung disease, atrial fibrillation, previous CABG, and previous PCI.

For the composite of MACCE and mortality outcomes, subgroup analyses were performed by sex (males and females), ACS type (STEMI, NSTEMI, and UA), and vessel severity (SVD, 2VD, and 3VD). Adjustment was performed for all the variables in the Cox Model 3 except for the variable defining each subgroup analysis.

All analyses were performed using R version 4.2.3 using packages “*survminer*” and “*survival*” and a two‐sided *p*‐value of less than .05 was considered statistically significant.

## 3 RESULTS

### 3.1 Baseline characteristics

Among the total of 14 528 patients ACS who underwent PCI from 2015 to 2021, glycemic status was available for 14 402 patients. Moreover, a further 720 patients did not have follow‐up data. Of the remaining 13 682 patients, 4697 (34.3%) were NG, 3151 (23%) were prediabetic, and 5834 (42.6%) were diabetic (Table 1). The mean ± SD age of NG patients was 61.4 ± 11.6 years, while pre‐DM patients had 62.5 ± 11.1 years and DM patients had a mean age of 63.8 ± 10.2 years (*p* < .001). Males were higher in NG patients (82%) which had a significant difference with pre‐DM (76.6%) and DM (62.9%) groups (*p* < .001). Overall, patients with DM had significantly higher rates of hypertension, dyslipidemia, heart failure, and peripheral vascular disease. Moreover, previous PCI, previous CABG, and history of CVA were significantly higher in patients with DM, followed by those with pre‐DM. In the lipid profile, diabetic patients had higher triglyceride and higher LDL‐C, while lower HDL‐C and lower hemoglobin. Mean LVEF means were 47.1 ± 8.5% in the NG group, 45.4 ± 9% in the pre‐DM group, and 44.7 ± 9.6% in the DM group, showing a significant difference (*p* < .001).

**Table 1:** Baseline characteristics of patients with ACS undergoing PCI by baseline glycemic status.

	Normoglycemic (*N* = 4697)	Prediabetes (*N* = 3151)	Diabetes (*N* = 5834)	*p* Value
Age (years)	61.4 ± 11.6	62.5 ± 11.1	63.8 ± 10.2	<.001
Sex (male)	3852 (82%)	2414 (76.6%)	3669 (62.9%)	<.001
BMI (kg/m^2^)	27.5 ± 4.2	28.4 ± 4.6	28.8 ± 4.6	<.001
Waist circumference (cm)	98.3 ± 10.1	100.6 ± 10.5	101.6 ± 10.7	<.001
Hypertension	2078 (44.2%)	1578 (50.1%)	3673 (62.9%)	<.001
Cigarette smoking	2338 (49.8%)	1401 (44.5%)	1901 (32.6%)	<.001
Dyslipidemia	2357 (50.2%)	1782 (56.5%)	4182 (71.7%)	<.001
Heart failure	94 (2%)	76 (2.4%)	204 (3.5%)	<.001
Atrial fibrillation	28 (0.6%)	24 (0.8%)	54 (0.9%)	.159
Valvular heart disease	78 (1.7%)	55 (1.7%)	103 (1.8%)	.914
Peripheral vascular disease	12 (0.3%)	4 (0.1%)	26 (0.4%)	.025
Chronic lung disease	103 (2.2%)	64 (2%)	145 (2.5%)	.820
Previous PCI	723 (15.4%)	481 (15.3%)	1150 (19.7%)	<.001
Previous CABG	378 (8%)	258 (8.2%)	683 (11.7%)	<.001
History of CVA	129 (2.7%)	71 (2.2%)	233 (4%)	<.001
Family history of CAD	942 (20%)	664 (21.1%)	1061 (18.2%)	.002
Opium consumption	870 (18.5%)	562 (17.8%)	692 (11.9%)	<.001
History of STEMI	260 (5.5%)	172 (5.5%)	338 (5.8%)	.761
History of NSTEMI	694 (14.8%)	347 (11%)	779 (13.3%)	<.001
History of UA	1767 (37.7%)	1033 (32.8%)	2040 (34.5%)	<.001
History of SA	109 (2.3%)	55 (1.7%)	115 (2%)	.186
LVEF (%)	47.1 ± 8.5	45.4 ± 9	44.7 ± 9.6	<.001
Total cholesterol (mg/dL)	156.8 ± 41	162.5 ± 41.7	156.1 ± 44.1	.188
Triglyceride (mg/dL)	140.5 ± 82.4	151.9 ± 97.8	169.1 ± 114.1	<.001
LDL‐C (mg/dL)	98.6 ± 34.4	102.5 ± 34.7	95.7 ± 35.3	<.001
HDL‐C (mg/dL)	39.1 ± 9.8	39.3 ± 9.4	38.6 ± 9.8	.006
FPG (mg/dL)	90.1 ± 7.2	109.5 ± 7.8	169.9 ± 64.9	<.001
HbA1C (%)	4.9 ± 0.3	5.5 ± 0.8	8.1 ± 2	<.001
Creatinine (mg/dL)	0.99 ± 0.49	0.98 ± 0.3	1.00 ± 0.55	.131
Hemoglobin (g/dL)	14.9 ± 1.7	14.9 ± 1.7	14.4 ± 1.9	<.001
Lesion length (mm)	25.2 ± 12.8	26.1 ± 13	26.3 ± 13.1	<.001
Preprocedure stenosis (%)	91.5 ± 8.1	92.1 ± 9.8	91.7 ± 9.1	.178
ACS Type	25.2 ± 12.8	26.1 ± 13	26.3 ± 13.1	<.001
STEMI	1395 (29.7%)	1308 (41.5%)	2225 (38.1%)	<.001
NSTEMI	987 (21%)	533 (16.9%)	974 (16.7%)	
Unstable angina	2315 (49.3%)	1310 (41.6%)	2635 (45.2%)	
TIMI flow				
0	1080 (23%)	973 (30.9%)	1590 (27.2%)	<.001
1	189 (4%)	110 (3.5%)	266 (4.6%)	
2	618 (13.2%)	431 (13.7%)	817 (14%)	
3	2810 (59.8%)	1636 (51.9%)	3161 (54.2%)	
Vessel severity				
Single vessel	1924 (41%)	1222 (38.8%)	1827 (31.3%)	<.001
Two vessels	1618 (34.4%)	1088 (34.5%)	1996 (34.2%)	
Three vessels	1145 (24.4%)	834 (26.5%)	2000 (34.3%)	
ACC/AHA category				
A	10 (0.2%)	3 (0.1%)	8 (0.1%)	<.001
B1	933 (19.9%)	556 (17.7%)	1004 (17.2%)	
B2	665 (14.2%)	472 (15%)	771 (13.2%)	
C	3088 (65.7%)	2119 (67.3%)	4050 (69.4%)	
PCI location				
Ostial	492 (10.5%)	348 (11%)	665 (11.4%)	.128
Proximal	1674 (35.6%)	1190 (37.8%)	2110 (36.2%)	
Nonproximal	2531 (53.9%)	1613 (51.2%)	3059 (52.4%)	

Abbreviations: ACC/AHA, American College of Cardiology/American Heart Association; ACS, acute coronary syndrome; BMI, body mass index; CABG, coronary artery bypass grafting; CAD, coronary artery disease; CVA, cerebrovascular accident; FPG, fasting plasma glucose; HDL‐C, high‐density lipoprotein cholesterol; LDL‐C, low‐density lipoprotein cholesterol; LVEF, left ventricular ejection fraction; MI, myocardial infarction; NSTEMI, non‐ST‐elevated myocardial infarction; PCI, percutaneous coronary intervention; SA, stable angina; STEMI, ST‐elevated myocardial infarction; TIMI, thrombolysis in myocardial infarction; UA, unstable angina.

Lesion length was significantly higher in DM patients while preprocedure stenosis (%) did not show any significant difference among the three groups. In all three groups, UA was the highest subtype of ACS. Finally, DM patients had a higher rate of three‐vessel disease, compared with two other groups.

### 3.2 Prediabetes and diabetes effect on MACCE and secondary outcomes

**Figure 1:**
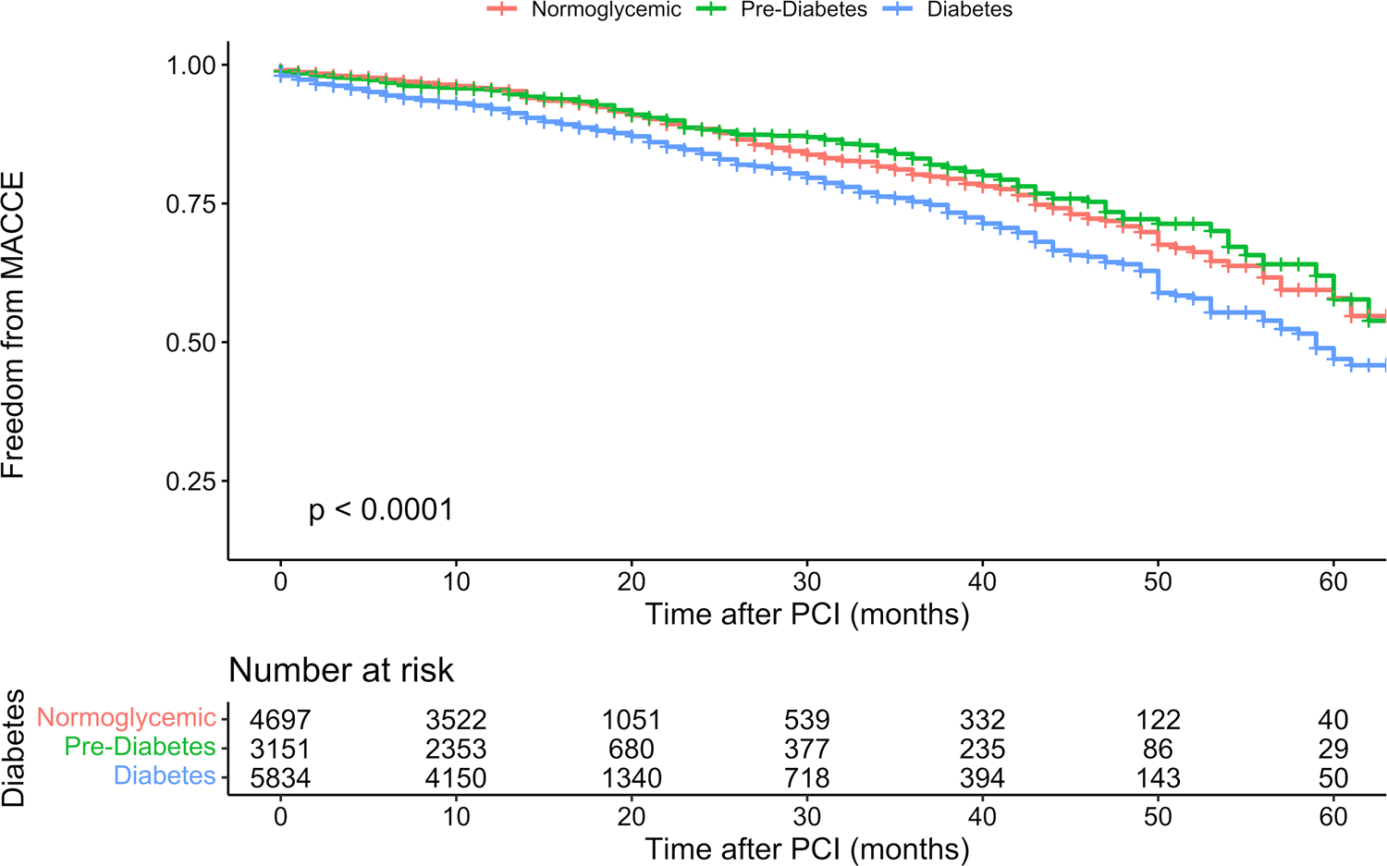
Freedom from MACCE composite outcome among normoglycemic, prediabetic, and diabetic patients. MACCE, major adverse cardiovascular and cerebrovascular events; PCI, percutaneous coronary intervention.

**Figure 2:**
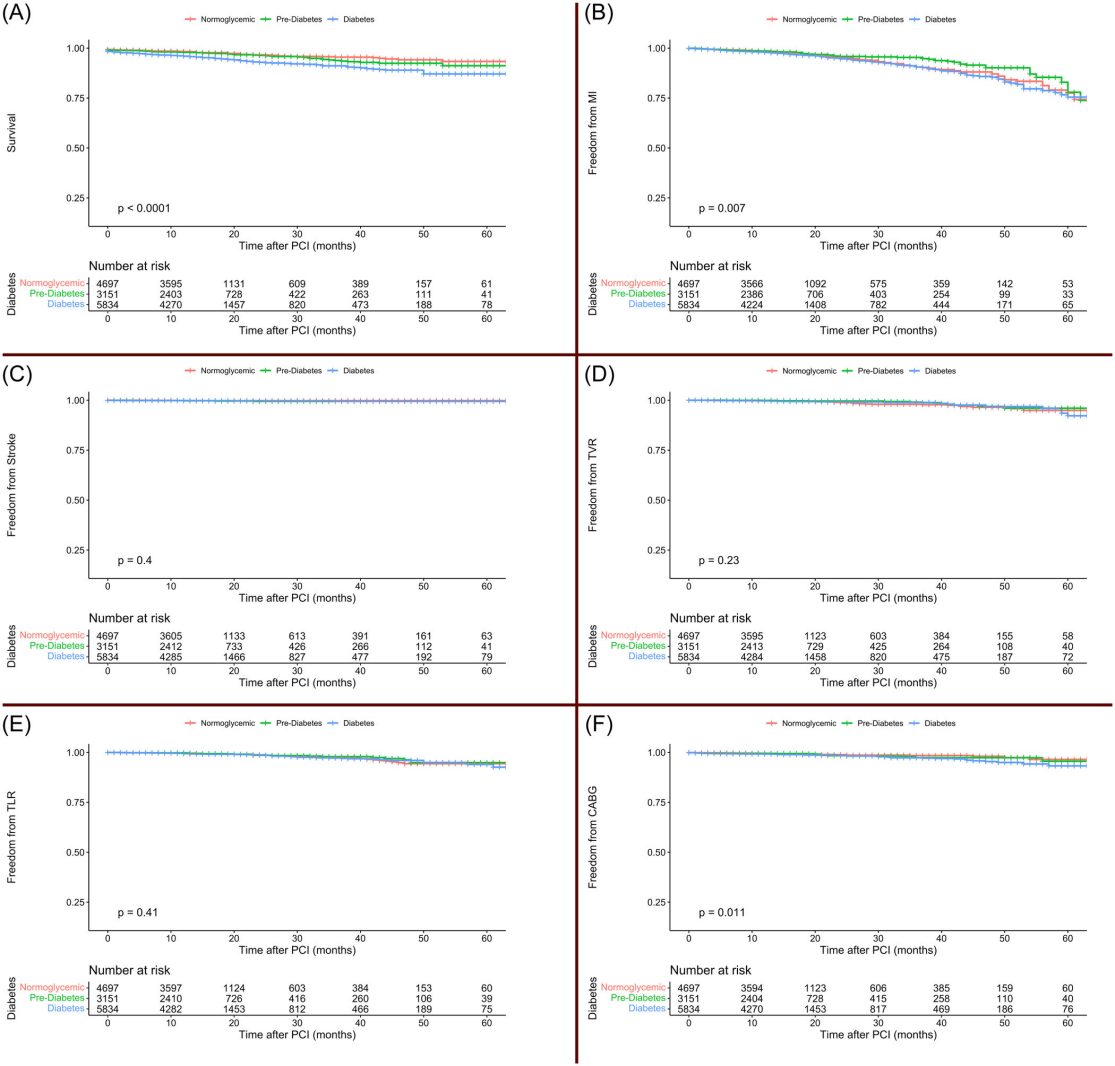
Freedom from MACCE components (A) mortality, (B) myocardial infarction, (C) stroke, (D) target vessel revascularization, (E) target lesion revascularization, and (F) CABG among normoglycemic, prediabetic, and diabetic patients. CABG, coronary artery bypass grafting; MACCE, major adverse cardiovascular and cerebrovascular events; PCI, percutaneous coronary intervention.

DM patients had significantly higher incidences of the composite of MACCE than NG patients across unadjusted, age‐ and sex‐adjusted, and fully adjusted Cox models (HR: 1.51, 95% CI: 1.34–1.71; 1.50, 95% CI: 1.33–1.70; and 1.22, 95% CI: 1.06–1.41, respectively) (Table 2). However, in pre‐DM patients, MACCE incidences were nonsignificantly lower than in NG patients in the three Cox models (Table 2). Figure 1 illustrates Kaplan–Meier curves for MACCE among the three glycemic status groups, and Figure 2A–F shows Kaplan–Meier curves for each of the MACCE components among the three glycemic status groups. Five‐year overall freedom from MACCE was 53% (95% CI: 48.9%–57.5%). This was 57.9% (95% CI: 51.4%–65.3%) in the NG group, 57.7% (95% CI: 48.8%–68.2%) in the pre‐DM group, and 47% (95% CI: 41%–53.8%) in DM group.

**Table 2:** Associations between glycemic status groups and primary and secondary outcomes across three Cox models.

	Event rate (%)	Unadjusted (Model 1)		Age‐ and sex‐adjusted (Model 2)		Fully adjusted (# Model 3)	
		HR (95% CI)	*p* Value	HR (95% CI)	*p* Value	HR (95% CI)	*p* Value
*Primary outcome*							
MACCE							
Normoglycemic	389/4697 (8.3%)	Ref	‐	Ref	‐	Ref	‐
Prediabetic	250/3151(7.93%)	0.96 [0.82–1.13]	.625	0.94 [0.80–1.10]	.422	0.95 [0.78–1.10]	.373
Diabetic	724/5834 (12.4%)	1.51 [1.34–1.71]	<.001	1.50 [1.32–1.70]	<.001	1.22 [1.06–1.41]	.006
*Secondary outcomes*							
All‐cause mortality							
Normoglycemic	111/4697 (2.36%)	Ref	‐	Ref	‐	Ref	‐
Prediabetic	90/3151 (2.86%)	1.22 [0.92–1.61]	.165	1.15 [0.87–1.52]	.326	1.15 [0.84–1.59]	.377
Diabetic	300/5834 (5.14%)	2.18 [1.76–2.72]	<.001	2.06 [1.65–2.57]	<.001	1.42 [1.08–1.86]	.012
Myocardial infarction							
Normoglycemic	156/4697 (3.32%)	Ref	‐	Ref	‐	Ref	‐
Prediabetic	83/3151 (2.63%)	0.80 [0.61–1.04]	.094	0.80 [0.62–1.05]	.111	0.83 [0.65–1.06]	.133
Diabetic	229/5834 (3.9%)	1.18 [0.96–1.45]	.110	1.26 [1.02–1.55]	.031	1.08 [0.89–1.30]	.460
Stroke							
Normoglycemic	7/4697 (0.15%)	Ref	‐	Ref	‐	Ref	‐
Prediabetic	9/3151 (0.28%)	1.93 [0.72–5.18]	.193	1.87 [0.69–5.02]	.216	2.05 [0.65–6.50]	.223
Diabetic	14/5834 (0.24%)	1.62 [0.65–3.86]	.298	1.61 [0.64–4.03]	.307	1.34 [0.44–4.12]	.608
Target vessel revascularization							
Normoglycemic	38/4697 (0.81%)	Ref	‐	Ref	‐	Ref	‐
Prediabetic	15/3151 (0.48%)	0.60 [0.33–1.09]	.091	0.59 [0.32–1.08]	.087	0.53 [0.27–1.04]	.065
Diabetic	42/5834 (0.72%)	0.88 [0.57–1.37]	.570	0.86 [0.54–1.34]	.495	0.84 [0.51–1.40]	.514
Target lesion revascularization							
Normoglycemic	43/4697 (0.92%)	Ref	‐	Ref	‐	Ref	‐
Prediabetic	25/3151 (0.79%)	0.87 [0.53–1.43]	.582	0.89 [0.54–1.45]	.631	0.86 [0.50–1.47]	.572
Diabetic	63/5834 (1.08%)	1.17 [0.80–1.73]	.419	1.24 [0.84–1.84]	.281	1.24 [0.80–1.93]	.332
CABG							
Normoglycemic	34/4697 (0.72%)	Ref	‐	Ref	‐	Ref	‐
Prediabetic	28/3151 (0.89%)	1.24 [0.75–2.04]	.406	1.24 [0.75–2.05]	.398	1.08 [0.64–1.84]	.760
Diabetic	76/5834 (1.3%)	1.79 [1.20–2.69]	.004	1.82 [1.20–2.75]	.004	1.68 [1.08–2.60]	.020

*Note*: Data are presented as hazard ratios (95% confidence intervals). # Model 3: adjusted for age, sex, LVEF, ACS type, vessel severity, TG, HDL‐C, LDL‐C, hypertension, WC, BMI, creatinine, hemoglobin, family history of CAD, smoking, opium use, past medical histories of STEMI, NSTE‐ACS (NSTEMI and UA), SA, heart failure, valvular heart disease, CVA, peripheral vascular disease, chronic lung disease, atrial fibrillation, previous CABG, and previous PCI.

Abbreviations: ACS, acute coronary syndrome; BMI, body mass index; CABG, coronary artery bypass grafting; CVA, cerebrovascular accident; HDL‐C, high‐density lipoprotein cholesterol; HR, hazard ratio; LDL‐C, low‐density lipoprotein cholesterol; LVEF, left ventricular ejection fraction; MACCE, major adverse cardiovascular and cerebrovascular events; PCI, percutaneous coronary intervention; SA, stable angina; STEMI, ST‐elevation myocardial infarction; TG, triglyceride; UA, unstable angina; WC, waist circumference.

DM patients had significantly higher incidences of all‐cause mortality than NG patients across the three Cox models (HR: 2.18, 95% CI: 1.76–2.72; 2.06, 95% CI: 1.65–2.57; and 1.42, 95% CI: 1.08–1.86, respectively). However, pre‐DM patients had nonsignificantly higher incidences of all‐cause mortality compared with NG patients (Table 2). Overall 5‐year survival rate was 90.1% (95% CI: 88.6%–91.7%), while this was 93.3% for the NG group (95% CI: 91%–95.7%), 91.2% for the pre‐DM group (95% CI: 88%–94.5%), and 87.1% for the DM group (95% CI: 84.6%–89.7%).

In the Cox model adjusted for age and sex, diabetic patients had a higher incidence of MI in comparison to NG patients (HR: 1.26, 95% CI: 1.02–1.55), but the association was not significant in the fully adjusted model. There were no significant associations between DM or pre‐DM and stroke, TVR, and TLR in comparison to NG patients. Incidence of CABG was significantly higher in DM patients in comparison to NG patients across the three Cox models (HR: 1.80, 95% CI: 1.20–2.69; 1.82, 95% CI: 1.21–2.75; and 1.68, 95% CI: 1.09–2.61, respectively).

### 3.3 Subgroup analyses by sex, ACS type, and vessel severity

**Figure 3:**
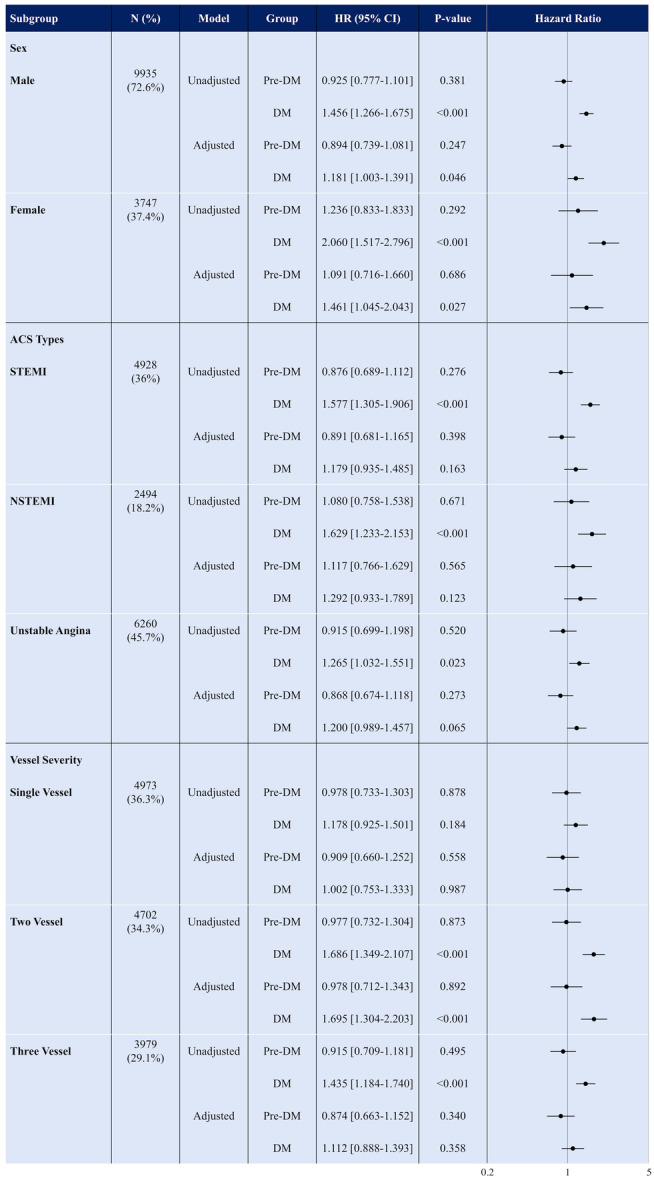
Association between glycemic status and MACCE outcome among subgroups by sex, ACS type and vessel severity. ACS, acute coronary syndrome; DM, diabetes mellitus; MACCE, major adverse cardiovascular and cerebrovascular events; NSTEMI, non‐ST‐elevated myocardial infarction; STEMI, ST‐elevated myocardial infarction.

The female subgroup showed a significant HR in adjusted (HR: 2.06, 95% CI: 1.52–2.80) and unadjusted (HR: 1.46, 95% CI: 1.04–2.04) (Figure 3). Among ACS types, diabetic patients with STEMI did not show any difference in the adjusted model (HR: 1.18, 95% CI: 0.93–1.48). Similarly, DM and pre‐DM patients with NSTEMI were not different in adjusted models (HR [95% CI]: 1.12 [0.77–1.63] and 1.29 [0.93–1.79]). Regarding vessel severity, patients with SVD did not show any difference in terms of diabetes and prediabetes. However, 2VD analysis for MACCE resulted in significant HR in patients with DM (HR: 1.69, 95% CI: 1.30–2.20) However, diabetic patients with 3VD did not show any difference in terms of MACCE incidence in unadjusted models (HR: 1.43, 95% CI: 1.18–1.74). All the analyses are shown in Figure 3. Kaplan–Meier curves for all these subgroup analyses are illustrated in Supporting Information: Figures 1–3.

Subgroup analyses for mortality in both males and females showed that in both unadjusted and adjusted models, diabetics, unlike prediabetics, had significantly higher HR in comparison with NG patients. In a subgroup of patients with UA, diabetic patients had a higher incidence in adjusted models (HR: 1.75, 95% CI: 1.07–2.87). Patients with SVD did not demonstrate any difference between NG, pre‐DM, and DM groups. On the other hand, diabetic patients with 2VD and 3VD diseases had higher mortality, compared with the NG group. Results of all adjusted and unadjusted survival analyses are depicted in Supporting Information: Figure 4, while the Kaplan–Meier curves are shown in Supporting Information: Figures 5–7.

## 4 DISCUSSION

We designed a cohort study to investigate the association between hyperglycemic states and the occurrence of MAACE, encompassing all‐cause mortality, cardiac death, TLR, and stroke, within a population of patients who underwent PCI. Patients with diabetes showed a significantly increased risk of the composite outcome of MACCE compared to NG patients in adjusted models. Patients with diabetes also had a greater risk of dying from all causes. Compared to NG individuals, prediabetic patients did not exhibit a statistically significant increased risk of MACCE or its components. The fact that prediabetic patients did not show elevated risk after PCI is of clinical importance. Subgroup analyses showed similar results to main analyses by type of ACS, severity of CAD, and gender.

Hyperglycemia can cause the incidence of the MACCE after PCI through some mechanisms such as increased oxidative stress, advanced glycation end products (AGEs), platelet activation, impaired endothelial function, and increased inflammatory response causing the development of atherosclerosis.^29^, ^30^, ^31^ AGEs are produced at an augmented rate, which causes oxidative stress and inflammation, which in turn causes endothelial dysfunction and accelerates atherosclerosis.^30^ Moreover, activation of protein kinase C isoforms also compromises nitric oxide signaling. The causes of hyperglycemia that contribute to the progression of atherosclerosis and CAD are increased flux through the polyol pathway, which results in oxidative stress, and activation of the hexosamine pathway, which makes inflammation and plaque instability.^31^, ^32^ Other factors include changes in coagulation, platelet reactivity, and microparticle release, hyperglycemia‐dependent microRNAs deregulation and loss of vascular repair capacities, and epigenetic‐driven transcription of genes that generate reactive oxygen species and promote inflammation.^33^, ^34^ When combined, these trailblazing discoveries suggest that generating mechanism‐based treatment plans is a practical way to reduce cardiovascular complications in people with diabetes.

A recent meta‐analysis of 17 studies demonstrated that the risks of all‐cause mortality, MI, and cardiac mortality were lower in the NG compared to the prediabetic patients at the longest follow‐up, with the mean of the follow‐up periods of the studies being 2.6 years. Moreover, patients with prediabetes had a lower risk of all‐cause mortality and cardiac mortality compared to patients with diabetes.^18^ In the HEART2D trial, patients with type 2 diabetes following PCI were randomized to receive prandial versus basal insulin. MACE did not differ between the groups at 1 year (11.0% vs. 12.3%, *p* = .33). An increased risk of MACE was linked to admission glucose levels of more than 180 mg/dL (HR: 1.58, 95% CI: 1.13–2.20).^35^ In comparison to NG patients, prediabetics had a twofold higher risk of death, MI, or repeat revascularization, according to a study of 2362 PCI patients, of whom 11% had prediabetes and 27% had diabetes. Intriguingly, this risk was not found to be significantly higher than that of diabetic patients.^21^ In contrast, our results demonstrated a nonsignificant difference in the incidence of the composite outcome of MACCE and each of its components, including all‐cause mortality, between NG and prediabetic patients. Compared to the prior studies, a significantly higher proportion of total included patients in our study, that is, 23% and 42.6%, were prediabetic or diabetic, respectively. The higher proportion of these two groups compared to the previous studies could render our results more representative. Diabetes on the other hand was identified as a risk factor for post‐PCI MACCE in an unadjusted model and in both models adjusted for possible confounding factors. In another study recruiting 242 prediabetics (36%) and 432 NG (64%) patients undergoing elective PCI, prediabetics had trends toward higher incidences of binary restenosis and late loss as short‐term post‐PCI outcomes, but similar rates of the composite outcome of MACCE, cardiac death, MI, and repeat revascularization in a 2‐year follow‐up period compared to 432 NG patients.^26^ The all‐cause mortality, however, was reported to be higher in the prediabetic group. A substantial rise in mortality was noticed in a meta‐analysis of 15 trials when admission glucose levels were higher than 140 mg/dL (risk ratio: 1.41, 95% CI: 1.18–1.69).^36^

When examining the collective results from studies comparing MACCE outcomes among individuals with prediabetes, diabetes, and those without diabetes, a consistent pattern emerges. These studies reveal that diabetes has a detrimental effect on long‐term outcomes following PCI compared to NG patients. However, there is much debate on the impact of prediabetes on the incidence of worse post‐PCI outcomes.

The enormous sample size of this study—more than 13 000 ACS patients receiving PCI—is one of its strongest points. This offers sufficient statistical power to identify variations in incident rates among groups with different glycemic statuses. The strength of this research lay in the detection of a 20% higher hazard for MACCE in diabetic compared to NG patients. An additional benefit is the prospective data collection from a national cardiac reference center, which guarantees uniform definitions and high‐quality data collection.

The study employed robust multivariable Cox regression analyses to account for relevant confounders, including age, sex, smoking, clinical risk factors, and the amount of angiographic illness. This makes it possible to separate the impact of glycemic status on results on its own. Predefined subgroup analyses were conducted according to sex, ACS type (STEMI, NSTE‐ACS), and CAD extent (single vs. multivessel). The prognosis of clinically different subgroups of ACS patients receiving PCI is revealed to change significantly depending on their glycemic status.

The current study's findings have several clinical applications. The most important clinical message might be the fact that prediabetic patients are not at higher risk of adverse events after PCI for ACS. This emphasizes that although lifestyle modifications should be advised to these patients, higher levels of care should still be given to those with confirmed diabetes. While patients with CAD have shared risk factors with those with metabolic syndrome,^37^ who are at higher risk of developing adverse events after PCI,^38^, ^39^, ^40^ close monitoring of prediabetic patients could be of high value due to the significant increase in MACCE rate from prediabetic to diabetic state.

Our study had several limitations. It is not possible to exclude residual confounding even after accounting for measured variables. Lack of information on drug adherence and postdischarge modifications may have an effect on long‐term results. Detection bias may be introduced if a clinical events committee does not systematically and independently adjudicate clinical events. A significant constraint is the short follow‐up period, with a median of approximately 1 year. A 3–5‐year follow‐up period might be more appropriate to fully clarify the long‐term effects of post‐PCI glycemic state. Additionally, the results only include glycemic status while a patient is hospitalized and do not take time variability or changes into account. HbA1c testing and serial glucose monitoring during follow‐up may provide light on the differences between the effects of acute deglycation and glucose control. A competing risks analysis of the components of MACCE was not conducted for the mortality outcome to take into consideration the interrelationships between the various causes of death. Additionally, the influence of hypoglycemia—which can negatively impact prognosis—was not examined in this study. Given the single‐center design and dearth of information on the distribution of races and ethnicities, generalizability may be restricted. Multicenter registries on a larger scale may improve generalizability.

## 5 CONCLUSIONS

This study found a substantial impact of diabetes on adverse cardiovascular outcomes in patients with ACS undergoing PCI. Patients with diabetes are at increased risk of MACCE and mortality. Despite prediabetic patients with ACS undergoing PCI having chronically elevated blood sugar levels, they did not show a significantly higher risk of MACCE compared to NG patients. This could give clinicians useful insight into giving care to prediabetic cases which are at the same risk as NG cases. Careful consideration to provide more intensive antiplatelet therapy, mitigation strategy for contrast‐induced acute kidney injury as a potential risk factor for MACCE, and the importance of hemodynamic support during high‐risk PCI in diabetes patients are important considerations for future research in the context of diabetes and cardiovascular care.

## AUTHOR CONTRIBUTIONS

*Study conception/data analysis/drafting the manuscript/revision*: Amir Hossein Behnoush, Saba Maleki, Alireza Arzhangzadeh, and Amirmohammad Khalaji. *Drafting the manuscript/revision*: Parmida Sadat Pezeshki, Zahra Vaziri, Zahra Esmaeili, Pouya Ebrahimi, and Haleh Ashraf. *Drafting and critical revision of the manuscript*: Farzad Masoudkabir, Ali Vasheghani‐Farahani, and Adrian V. Hernandez. *Study conception/drafting the manuscript/critical revision of the manuscript*: Kaveh Hosseini and Mehdi Mehrani. All authors read and approved the final manuscript.

## CONFLICT OF INTEREST STATEMENT

The authors declare no conflict of interest.

## Supporting information

Supporting information.

## Data Availability

The data used in this study will be made available upon reasonable request from the corresponding author.
