# Supplementary material for: Prediabetes and major adverse cardiac events after acute coronary syndrome: An overestimated concept
Source: Clin Cardiol. 2024 Apr 1;47(4):e24262. doi: 10.1002/clc.24262 (PMC10983809; doi:10.1002/clc.24262)
Supplement: Supplementary file 1 — Supporting information. [file CLC-47-e24262-s001.docx]

***Supplementary Figures***

***
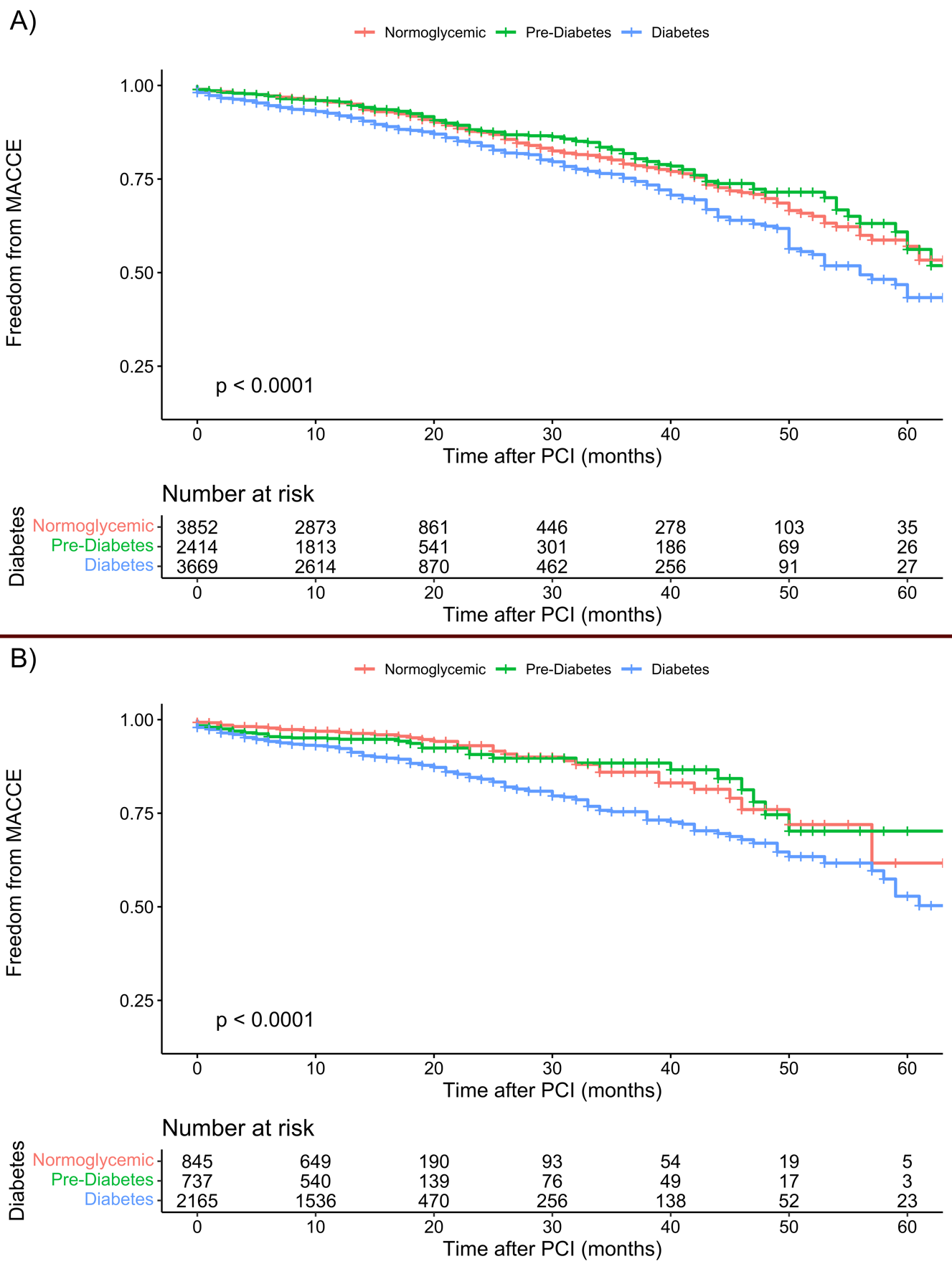
***

***Supplementary Figure 1.*** *Kaplan-Meier curves of the composite of MACCE among normoglycemic, prediabetic, and diabetic patients in A) males and B) females*

*
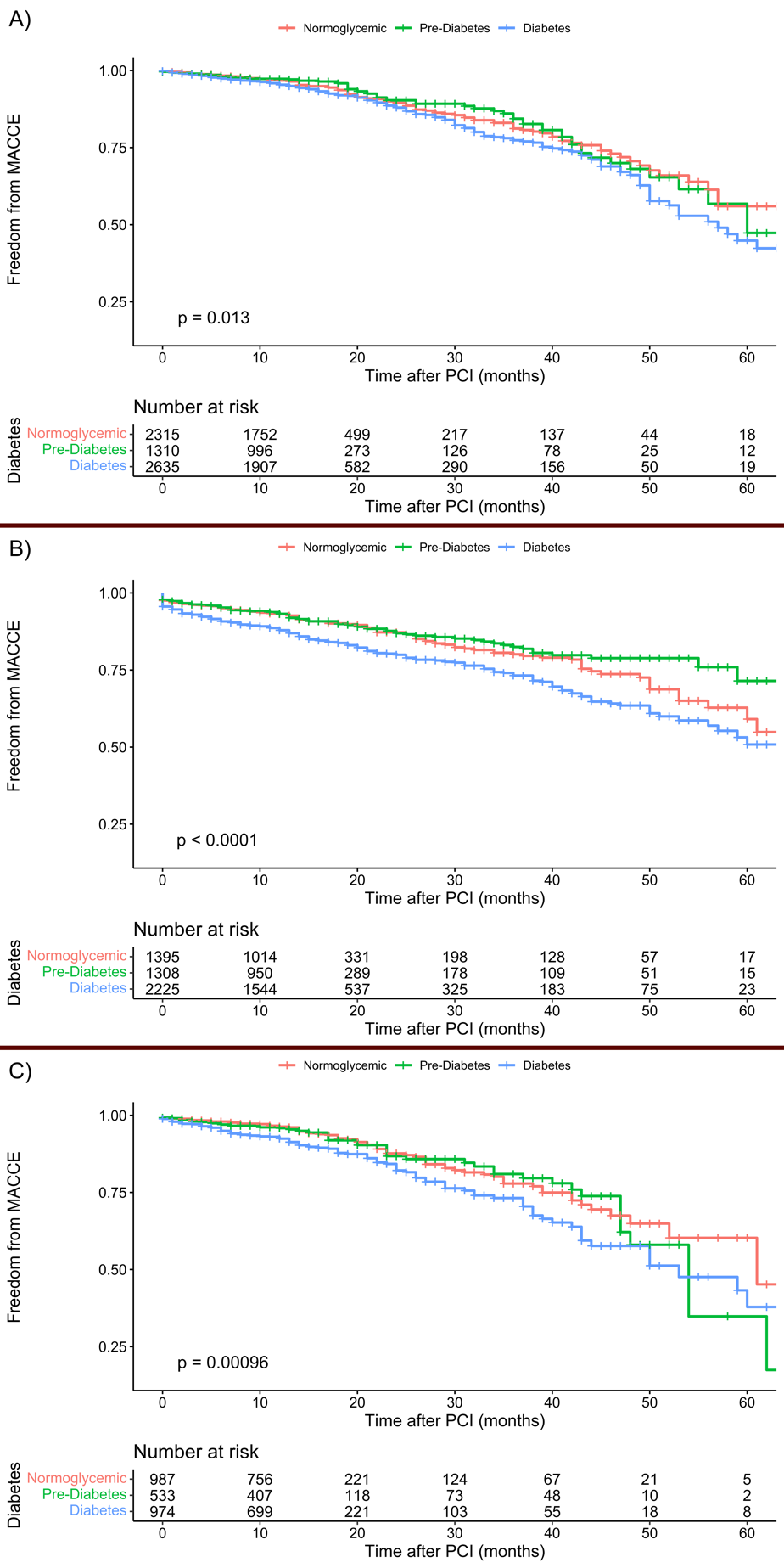
*

***Supplementary Figure 2.*** *Kaplan-Meier curves of the composite of MACCE outcome among normoglycemic, prediabetic, and diabetic patients with A) unstable angina, B) STEMI, and C) NSTEMI*

*
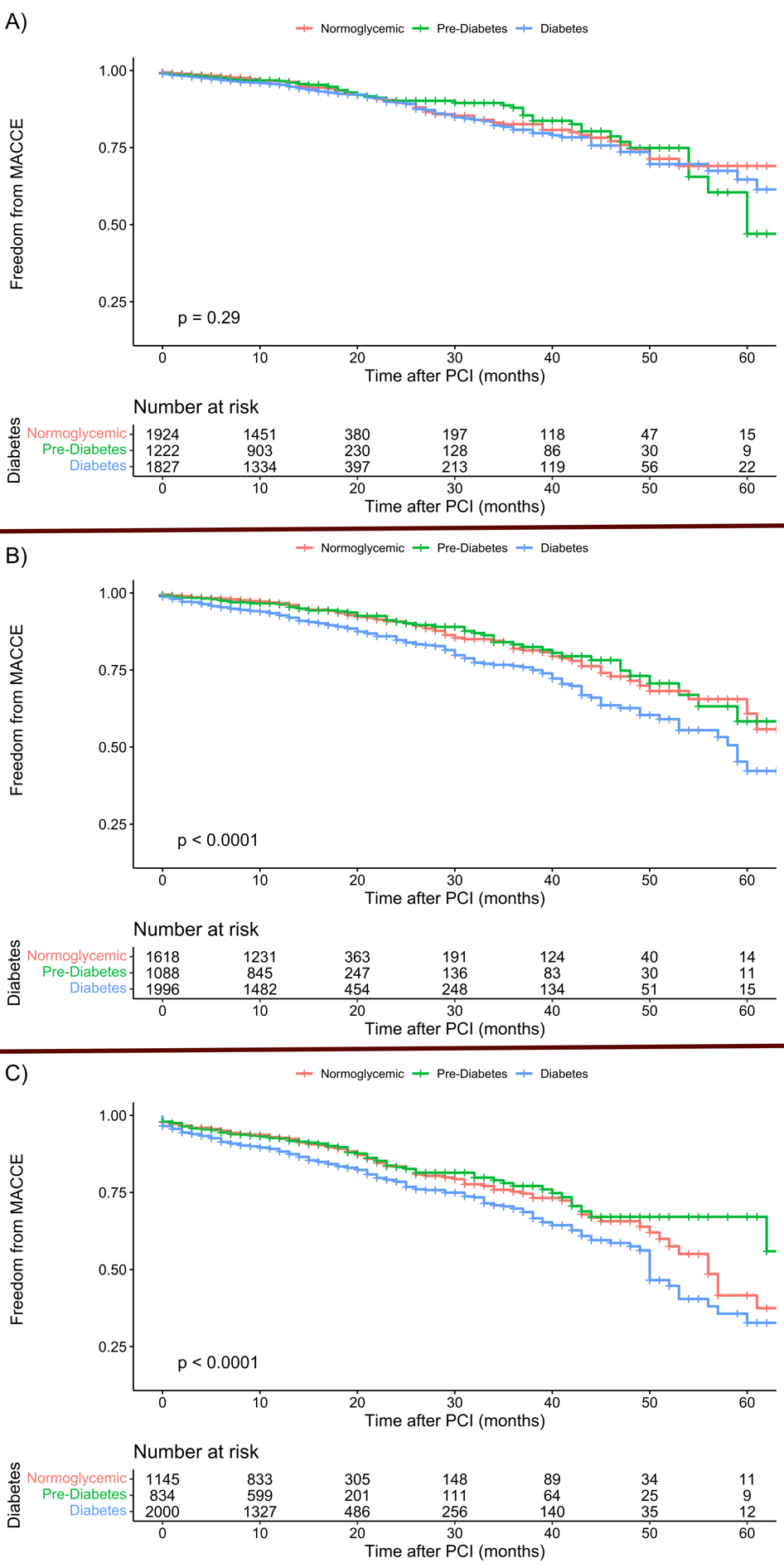
*

***Supplementary Figure 3.*** *Kaplan-Meier curves of the composite of MACCE among normoglycemic, prediabetic, and diabetic patients with A) single-vessel disease, B) two-vessel disease, and C) three-vessel disease*

*
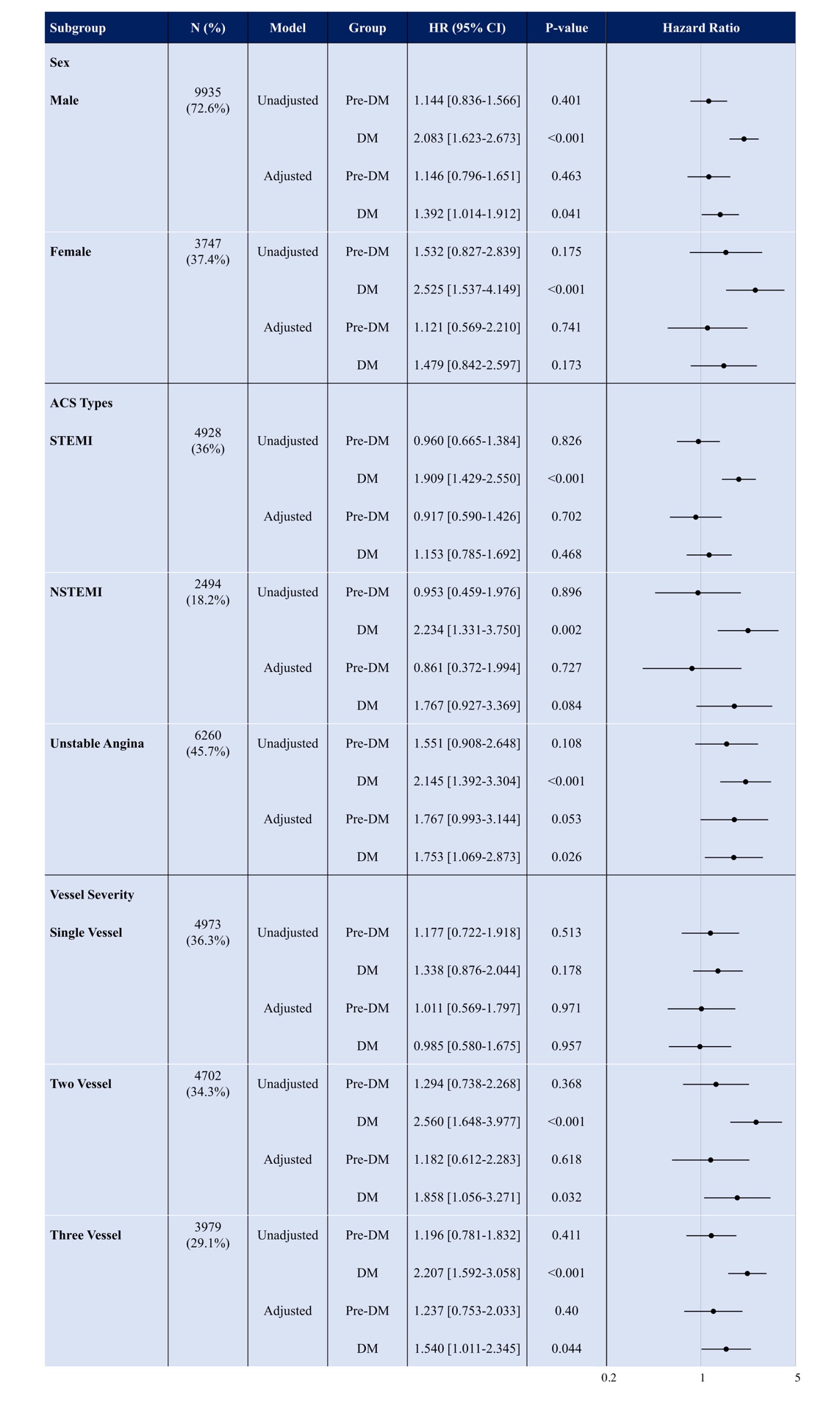
*

***Supplementary Figure 4.*** *Subgroup analyses by sex, ACS types and vessel severity for all-cause mortality. The reference is the normoglycemic group.*

*
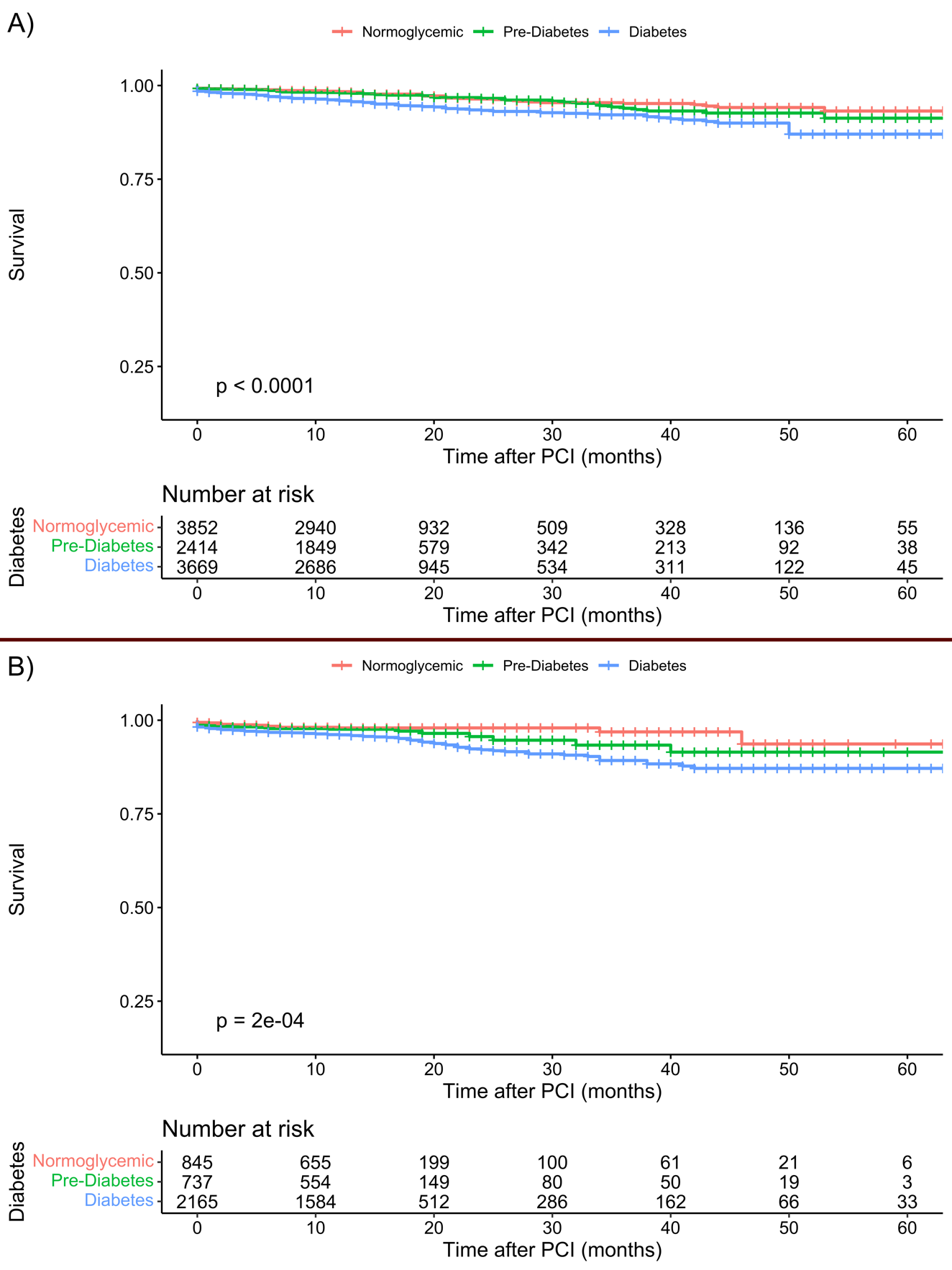
*

***Supplementary Figure 5.
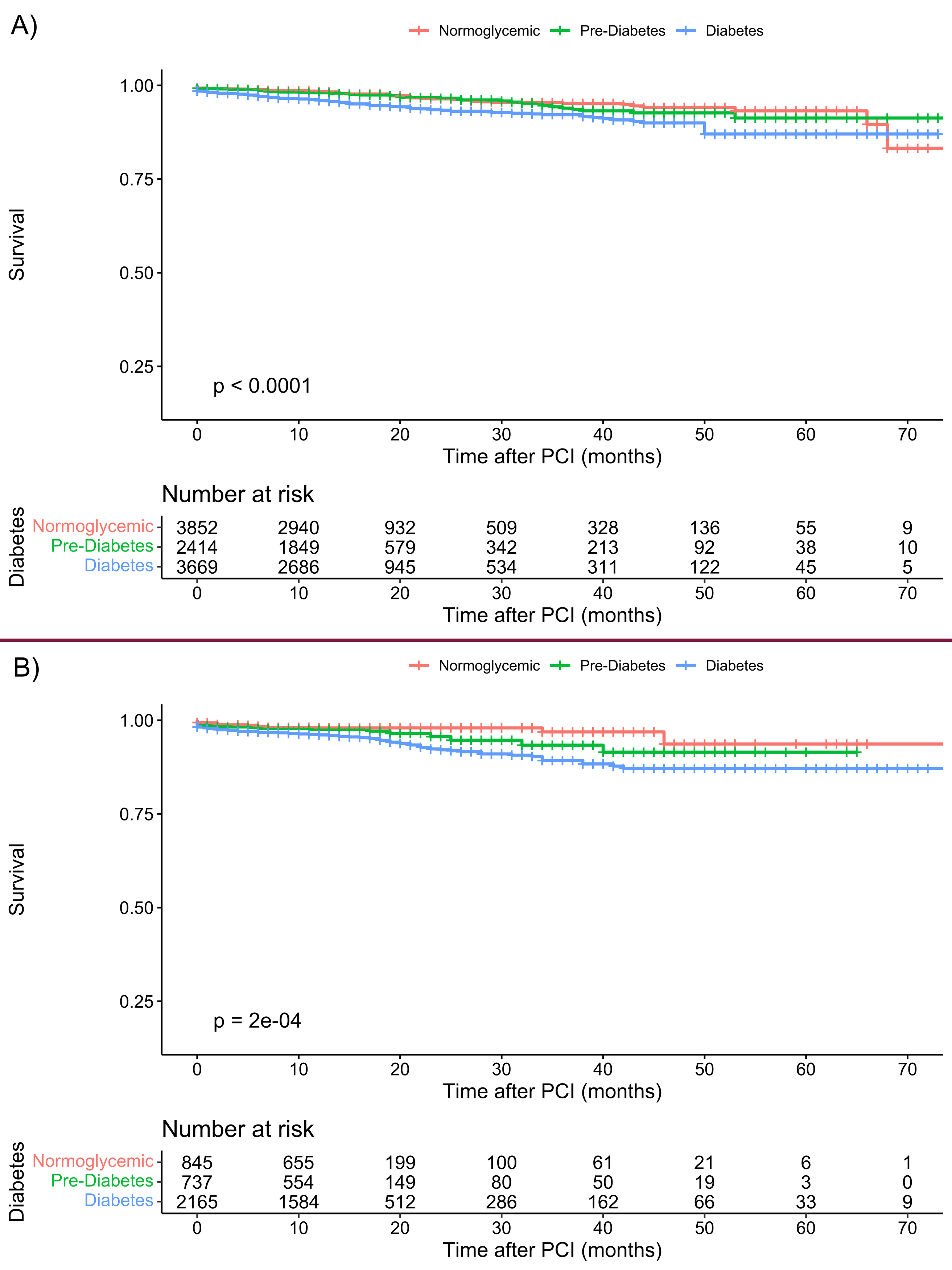
*** *Kaplan-Meier curves of all-cause mortality among normoglycemic, prediabetic, and diabetic patients in A) males and B) females*

*
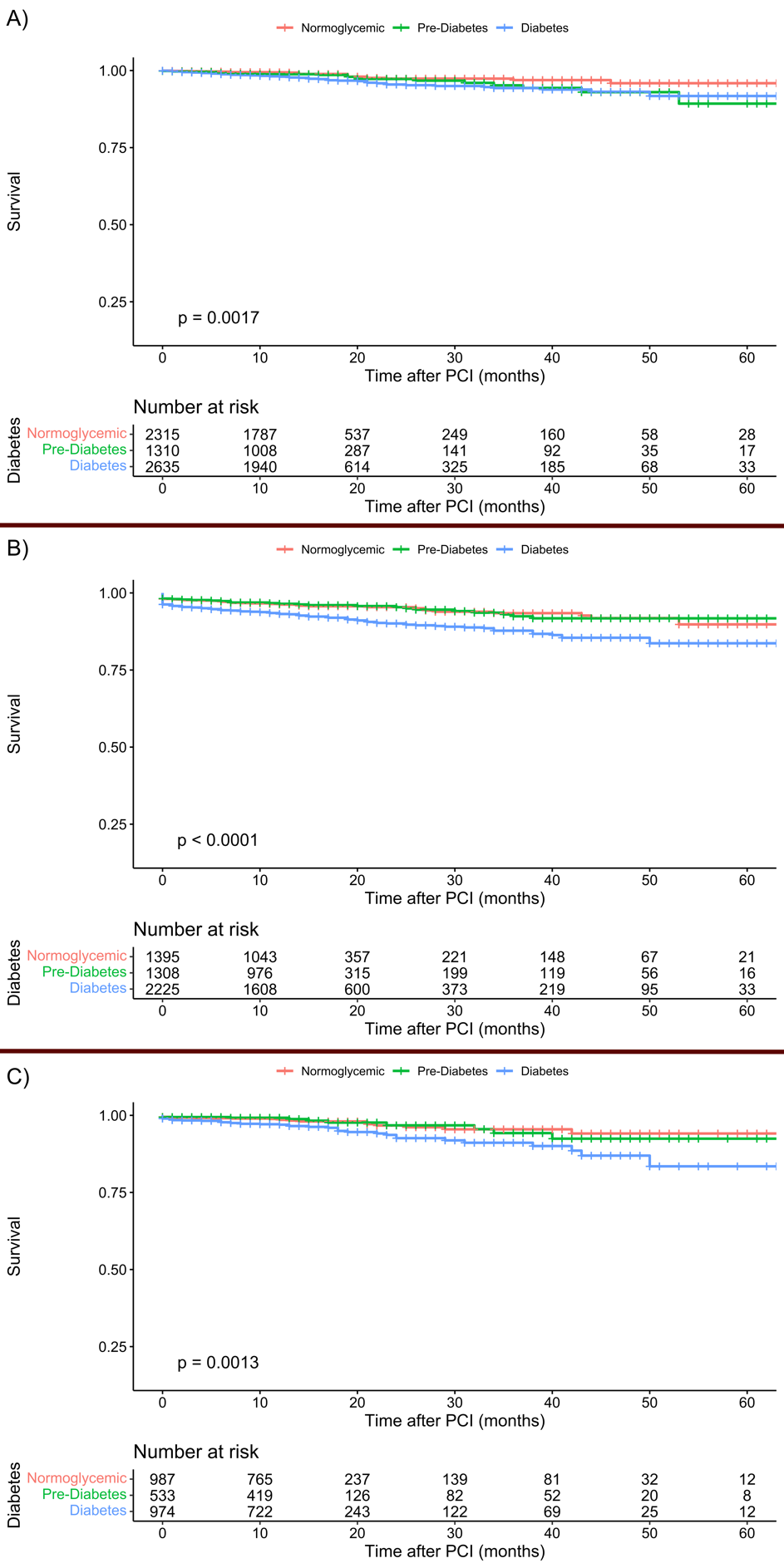
*

***Supplementary Figure 6.*** *Kaplan-Meier curves of all-cause mortality among normoglycemic, prediabetic, and diabetic patients with A) unstable angina, B) STEMI, and C) NSTEMI*

*
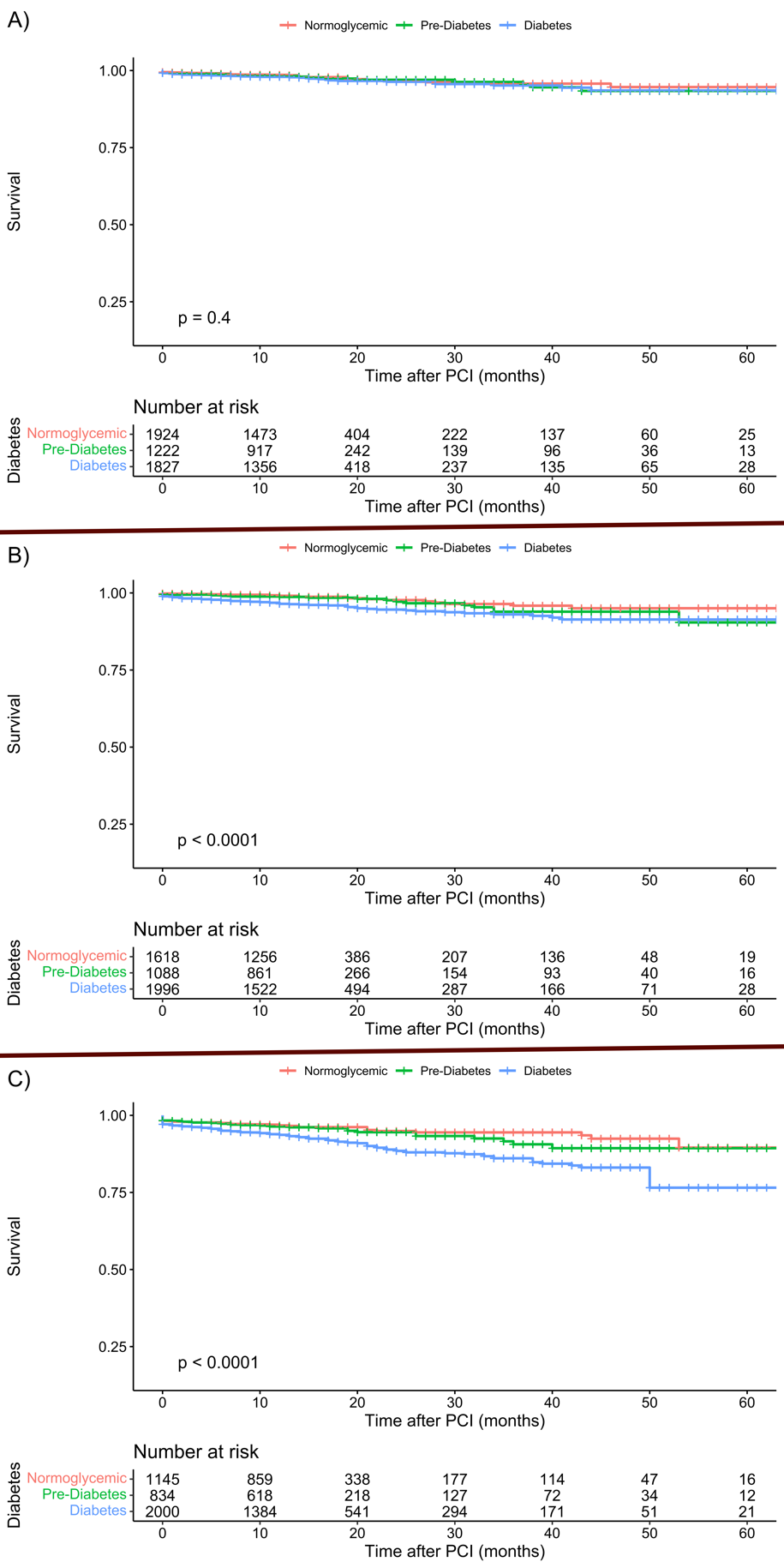
*

***Supplementary Figure 7.*** *Kaplan-Meier curves of all-cause mortality among normoglycemic, prediabetic, and diabetic patients with A) single-vessel disease, B) two-vessel disease, and C) three-vessel disease*
